# Investigating key drivers influencing AI-based detection and identification of plants

**DOI:** 10.1371/journal.pone.0342712

**Published:** 2026-03-02

**Authors:** Andréanne Charron, Adèle Julien, Joseph R. Stinziano, Marie-Claude Gagnon

**Affiliations:** 1 Genotyping-Botany Laboratory, Canadian Food Inspection Agency, Ottawa, Ontario, Canada; 2 Plant Health Risk Assessment Unit, Canadian Food Inspection Agency, Ottawa, Ontario, Canada; 3 Plant Data, Analytics and Modelling Unit, Canadian Food Inspection Agency, Ottawa, Ontario, Canada; Old Dominion University, UNITED STATES OF AMERICA

## Abstract

In recent years, AI-driven platforms have transformed citizen science by collecting and generating valuable records of living organisms for monitoring biological data. Many applications utilize visual similarity and geospatial information to identify species based on photographs. This study investigates how location impacts plant identifications made by iNaturalist, particularly in detecting invasive alien plants (IAP) that are not established in an area. We also compare the accuracy of iNaturalist and PlantNet while exploring potential biases. To assess iNaturalist’s taxonomic accuracy under varying location parameters, specimens of plants that are native and naturalized in Ontario, termed “established plants” for the purpose of this study, were collected and photographed (n = 61) and photographs of plants from Canada’s regulated pest list, which are either not present or have a very limited distribution in Canada, termed “outsider plants” for the purpose of this study were exported from iNaturalist and GBIF (n = 402). We used photographs of the established plants to compare taxonomic accuracy between applications, considering factors such as plant families, distribution status, and visible parts. A scoring system was established, and a cumulative linked mixed model was applied to analyze taxonomic accuracy. Our findings reveal that restricting location significantly hinders iNaturalist’s ability to identify IAP, highlighting the potential for missed detections. While sample size limitations prevented a robust comparison between applications, we also found significantly lower identification accuracy for species in the Poaceae family and for photographs featuring only leaves. Ultimately, recognizing the influence of location is essential for effectively monitoring IAP and leveraging iNaturalist as a tool for early detection.

## Introduction

Biological invasions of invasive alien plants (IAP) have become an increasingly difficult problem in our modern world. Fueled by growing human population size and trends in globalization, many IAP have been able to overcome biogeographical barriers and establish in many regions of the world [1,2]. Recent analysis confirms that IAP have significant and widespread economic, ecological, cultural, and human health impacts [3–6]. The removal and eradication of invasive species is expensive, making proactive strategies such as risk assessment, prevention, surveillance, and early detection a less costly and more effective long-term approach [7–9].

Surveillance is a crucial step in early detection of invasive species, and requires coordination among scientists, researchers, land managers, government officials and citizens [8,10,11]. Typical surveillance methods often require specialized personnel and equipment, which can be costly and logistically challenging. Due to these limitations, recent efforts have pivoted towards public engagement initiatives, such as citizen science projects, as an effective way of detecting invasive species [10,12–15]. Citizen science is a collaborative research methodology in which projects are designed and led by researchers, while members of the general public contribute to data collection, classification, or analysis [15]. It offers the advantage of gathering extensive data across large areas, including regions often overlooked by scientists, such as urban settings [16,17]. This method of data collection has been used in a variety of projects such as biological monitoring, conservation, population modeling, invasive species distribution modeling and the detection of new invaders [12,16,18,19].

There are numerous citizen science projects worldwide, such as eBird (https://ebird.org/home), Flora Incognita (https://floraincognita.com/), iNaturalist (https://www.inaturalist.org/) and PlantNet (https://identify.plantnet.org/), which use artificial intelligence to propose identifications and taxonomically classify observations made by users [20].The concept of using artificial intelligence for plant identification began in the 1980s when Guyer and colleagues [21] analyzed plant characteristics and proposed the potential of machine learning for this purpose. Since then, artificial intelligence has become widely used for various applications and serves as a valuable tool for plant identification [12,22]. Commonly used applications such as iNaturalist and PlantNet implement artificial intelligence classification systems using databases of vouchered observations combined with spatiotemporal data to weigh the computer vision results and suggest an identification [23,24]. These platforms implement verification processes to improve identification accuracy. On iNaturalist, observations can become ‘research grade’ when identifications to the genus or species level are confirmed by at least two users [23]. PlantNet uses a voting system, requiring a sufficient number of votes to validate an identification [24]. Once verified, research grade observations from iNaturalist or validated identifications from PlantNet are aggregated into larger databases such as the Global Biodiversity Information Facility (GBIF) [25], which as of November 17, 2025, contains over 3 billion occurrence records.

When monitoring invasive species, accurate identification is crucial in determining the best course of action to prevent the establishment of any potential invaders. With the growing use of data produced by such applications, precise identification is important in detecting invasive species. Recent studies have tested the accuracy and reliability of such platforms, focusing on the proposed identifications of posted observations [16,26–30]. Such studies have shown great success in some taxa, such as herpetofauna studies [29], while more difficult taxa such as lichens have shown much more frequent misidentifications [26]. Evaluation of such platforms is crucial since data collected on these applications is frequently used for biodiversity management and conservation efforts [16,31].

The iNaturalist application suggests taxa that are both visually similar and likely to be found nearby, provided that such taxa are expected in the area. Alternatively, iNaturalist will show visually similar taxa regardless of what is seen nearby if there are no visually similar taxa anticipated in the area [32]. In 2022, we investigated the effect of location precision on the suggested identification provided by computer vision and assessed its accuracy to understand how it can influence the detection of IAP introductions on iNaturalist. On September 21^st^, 2023, iNaturalist issued a statement acknowledging issues with the Geomodel, clarifying that by default only a subset of nearby suggestions is presented [33]. Prior to this date, computer vision suggestions relied on a gridded version of the raw observations. In observations up to mid-2023, the “Expected Nearby Map” predicted by the Geomodel has been used to apply the nearby label. They reported that the correct suggestion was included among the nearby subset in 94% of observations, both for the gridded version and for the Geomodel. The underlying statistics, calculations and type of species used to make this analysis were not shared. Although examples of unusual observations were provided along in their statement, primarily involving insects and birds, the extent to which incorrect suggestions are made with new introductions remains unclear, especially in plants.

To address this gap, our study evaluates the taxonomic accuracy of AI-generated plant identifications from a user perspective, focusing on the accuracy of the suggestions as they appear in practice rather than the internal mechanisms of the models. Specifically, we conducted a comparative assessment of iNaturalist and PlanNet to identify potential biases related to geographic precision, taxonomic group, morphological features, and species distribution status. This investigation was guided by five research questions: 1) Does the accuracy of iNaturalist’s computer vision suggestions decline with increasingly precise location data for plant species not typically present in the surrounding environment? 2) Does iNaturalist and PlantNet differ in their overall identification accuracy under comparable testing conditions? 3) Does identification accuracy vary among plant families within each application? 4) Does the inclusion of reproductive structures (e.g., flowers or inflorescences) in single-image observations improve the accuracy of computer vision suggestions? 5) Are long-established introduced species identified as accurately as native plants? Together, these research questions aim to clarify the reliability of automated identification tools and provide insights for both monitoring programs and general users, with a particular emphasis on the early detection of IAP introductions as iNaturalist continues to refine its models.

## Methods

### Sampling of established plants

In 2022, 61 plants were randomly collected from four locations within the Ottawa region of Ontario, Canada: Ottawa Laboratory, Nepean (45.287659, −75.770410), Sandy Hill, Ottawa (45.418558, −75.676766), Experimental Farm, Central Park (45.384524, −75.709887) and Calabogie (45.304705, −76.715290). Ottawa is the capital city of Canada, with an area of 2,778 km^2^, located in the province of Ontario with an area of more than 1 million km^2^. Collection sites were restricted to areas where invasive species are usually present, such as disturbed sites, roadsides, and ditches [34,35]. Collection sites included both public and private land. Sampling on private properties was conducted with authorized access, and no permits were required. Three pictures were taken of most of the collected plants at the collection site to display different parts of the plant (leaf, inflorescence, and whole plant). All pictures were taken with an iPhone 12. Plants were identified to species level through independent morphological testing by trained botanists (the authors AC and AJ) and categorized according to plant family, as well as to distribution status in Ontario: native or introduced. The distribution status was determined using Vascan [36]. In Vascan, taxa that have established following European colonization are labeled as introduced. The collected plants belong to the “established plants” group in the study.

### Citizen science records of outsider plants

Plant species from the Canadian Food Inspection Agency (CFIA)’s list of regulated pest plants [37] were selected to represent the “outsider plants” group. These plants are known for their invasive traits and are either not established in Canada or have a very limited distribution. A “collection project” was created on iNaturalist on 2022/09/07 to export user observations of the outsider plants [38]. A set of observations was exported on 2023/01/31 for *Echium plantagineum* L., *Pueraria montana* (Lour.) Merr., *Microstegium vimineum* (Trin.) A. Camus, *Aegilops cylindrica* Host, and *Solanum elaeagnifolium* Cav. These species were chosen based on ease of identification and observations were restricted to North America. A randomized dataset of approximately 50 observations for each species were exported. For each observation, the pictures were analyzed by the authors AC and AJ and sorted into “correctly identified”, “incorrectly identified”, and “not possible to identify”, with only the “correctly identified” kept for the taxonomic accuracy assessment. Permission to use pictures with “All Right Reserved” copyright was requested of users, and only those that were granted permission were included in the testing (Table I in S1 Data). In late 2023, a second round of observations was exported to increase statistical power to include *Alopecurus myosuroides* Hudson*, Eriochloa villosa* (Thunberg) Kunth, as well additional observations of *E. plantagineum* and *P. montana*. New observations were randomly selected from the occurrence data on 2024/01/08 in ‘Beyond a diagnostic tool: Validating standardized Mahalanobis distance as a species distribution model for invasive alien species in North America’ from Stinziano et al. [19] and on 2024/01/09 for *E. plantagineum* from GBIF [39]. These observations were primarily from iNaturalist in North America, except for *A. myosuroides* and *E. villosa*, which include records outside North America due to insufficient data reported at the time in North America. The observations retrieved from Stinziano et al. had already been verified by author AC and sorted according to the same three identification categories noted above. Data for *E. plantagineum* was exported from GBIF, as this has become the recommended method for using iNaturalist data. Observations of *P. montana* and *E. plantagineum* were sorted following the same method. Only the most useful picture for morphological identification was used in the taxonomic accuracy assessment. All pictures included are research grade. The sorting resulted in 402 samples: *A. cylindrica* (n = 37), *A. myosuroides* (n = 100), *E. plantagineum* (n = 44), *E. villosa* (n = 100), *M. vimineum* (n = 35), *P. montana* (n = 46) and *S. elaeagnifolium* (n = 40).

### Taxonomic accuracy assessment

To assess the accuracy of computer vision-based identifications, we conducted a structured assessment focusing on the suggestions provided by the applications from a user perspective, rather than the internal mechanisms of the models. Pictures from the established and outsider groups described above were uploaded to both applications over two trial periods (2022–2023 and 2023–2024). The established group consisted of species with long-established populations, while the outsider group included species not present or with a very limited distribution in Canada. This design enabled assessment of the effect of location precision on identification accuracy, particularly for plants not typically present in a given area. It also allowed comparison of accuracy between native species and long-established introduced species within the established group.

For iNaturalist, an account was created, and pictures were uploaded via the “Upload” page. Selecting the “Species name” field triggered the application’s AI to provide either a single suggestion or a ranked list. Pictures were uploaded to PlantNet via the web interface, also with manual location entry, and the application returned a ranked list of top species suggestions. For both applications, only the first suggestion was recorded, here referred to as “assessment score”. Recording the first suggestion mirrors typical user reliance on the AI recommendations and provides a standardized measure of accuracy. Each suggestion was scored against botanist confirmed identifications using a scale: correctly identified (0), species-level error (1), genus-level error (2), family-level error (3), kingdom-level error (4) or no suggestion-level error (5).

To investigate the effect of location on taxonomic accuracy, location settings were systematically varied, and initial uploads were discarded between tests to prevent carryover effects. In the first trial period, the location in iNaturalist was set to Ottawa (OTT) or Ontario (ON), while the location in PlantNet was set to Canada (CAN) and World Flora, as finer-scale precision was not available. In the second trial period, PlantNet refined its location settings, and “Eastern Canada” was selected to best reflect the collection sites of the established plants. Simultaneously, No Location (NL) was included for iNaturalist alongside the other settings. NL is roughly comparable to World Flora on PlantNet and allowed us to assess AI suggestions in the absence of the location effect. Including this setting also ensured that location effects did not influence other factors being tested, such as differences in accuracy between applications, plant families, and species distribution status.

Due to platform constraints on PlantNet, the outsider group pictures were uploaded exclusively to iNaturalist. For some species (e.g., *P. montana*), available pictures included only singular leaves, which did not meet PlantNet’s requirements for multiple pictures. Although, PlantNet can provide suggestions from a single picture, limited training data for these species may have prevented this option at the time of testing. Consequently, only data from the second trial period tested on iNaturalist were used to evaluate the effect of location for both plant groups, making PlantNet data irrelevant for this specific research question. Similarly, the data from the first trial period were excluded from analyses related to location, application, plant family, and species distribution status effects as the NL setting was not used.

**Fig 1:**
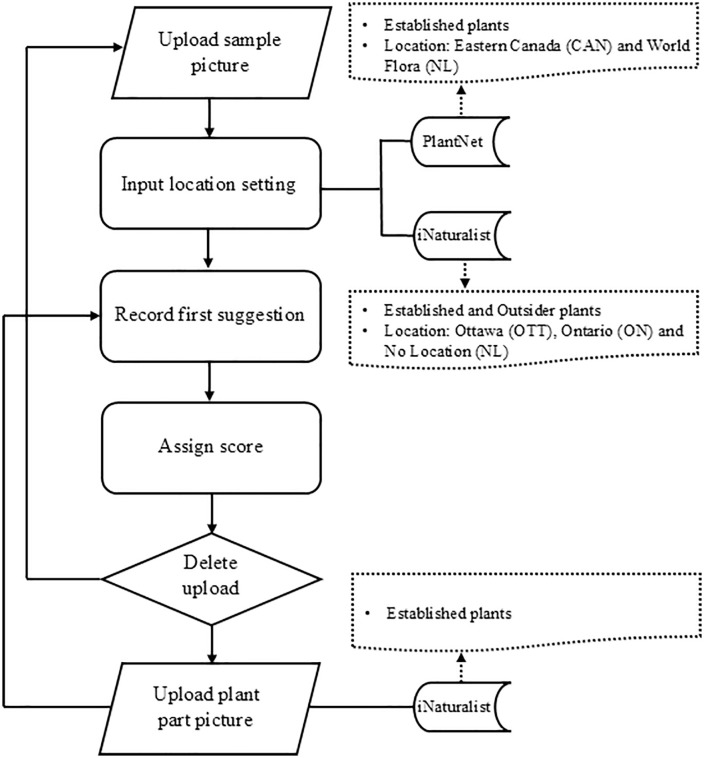
Workflow for testing AI-based plant identifications across applications. The diagram summarizes the stepwise procedure used to assess identification suggestions obtained from iNaturalist and PlantNet across different locations settings and plant parts.

To examine the influence of plant morphology, a subgroup of 29 samples from the established group was used to test if different parts of a plant (e.g., leaf, inflorescence, whole plant) can impact identification accuracy. Each image was uploaded individually per sample to iNaturalist with no specified location, and the first suggestion was recorded. Pictures were only uploaded during the first trial. For the same reason as the outsider group pictures, they were only uploaded to iNaturalist. Pictures were cropped when necessary to only highlight the relevant plant part under evaluation, due to challenges in achieving precise framing in the field. All pictures from the established group were deposited in a Zenodo repository. Fig 1 provides a summary of the workflow for testing AI-based plant identifications across applications.

### Statistical analysis

The data collected for statistical analysis included assessment scores from both the outsider and established groups during the second trial period (iNaturalist; n = 463) to test the effect of location. Assessment scores from the established group during the second trial period (iNaturalist and PlantNet; n = 61) were used to test the effects of tool type, family and distribution status. Finally, assessment scores from the established group during the first trial period (iNaturalist; n = 29) were used to test the effect of plant parts. Table 1 provides a summary of the data collected for statistical analysis, the models applied, and the associated predictor variables and interactions, where applicable for each research question.

**Table 1:** Summary of the models used and relevant parameters to evaluate the taxonomic accuracy score in response to the effects tested.

Effect tested	Data set	Sample size	Tool	Predictor variable	Plant group	Model	Random effect	Interaction
**Location**	2^nd^ Trial period	n = 463	iNaturalist	Ottawa Ontario No Location	Established n = 61	Clmm2	LabID	N/A
					Outsider n = 402	Clmm2	CatalogID	N/A
						Polr	N/A	N/A
**Family**	2^nd^ Trial period	n = 61	iNaturalist PlantNet	Asteraceae Poaceae Other	Established	Clmm2	LabID	Tool type
**Distribution** **status**	2^nd^ trial period	n = 61	iNaturalist PlantNet	Introduced Native	Established	Clmm2	Lab ID	Tool type
**Plant parts**	1^st^ Trial period	n = 29	iNaturalist	Inflorescence Leaves Whole plant	Established	Clmm2	LabID	N/A

1^st^ trial period: 2022–2023 taxonomic accuracy assessment, 2^nd^ trial period: 2023–2024 taxonomic accuracy assessment. clmm: cumulative link model, clmm2: cumulative link model version 2, polr: proportional odds logistic regression. N/A: not applicable.

The statistical language R, version R 4.3.2 [40] was used to build and run the analysis. The Cumulative Link Mixed Model version 2 (clmm2) from the ordinal package [41], fitted with the adaptive Gauss-Hermite quadrature approximation, was used to evaluate the taxonomic accuracy score, the outcome variable “assessment”. Only the assessment scores 0–3 were used to run the analysis. The Gauss-Hermite quadrature approximation was run with 7 quadrature points. This model was selected as the outcome variable is of ordinal nature and allows the inclusion of random effects and testing of interactions between predictor variables. Random effects (“Lab ID” for the established group and “Catalog ID” for the outsider group) accounted for group-level variability.

The independent variables were entered as fixed factors: location, tool type, family, and distribution status effect. The interaction between the predictor variables, plant family and distribution status with the tool type (iNaturalist and PlantNet) were also included. The pnorm function was used to find the p-values for a right-tail test. The predict function was used to get predicted probabilities for each taxonomic accuracy score. Plotting was performed using ggplot2 [42] to plot the predicted probabilities.

To estimate if the proportional odd assumption (POA) is true, a pairwise comparison of the estimated marginal means between the levels of the predictor variables for each model was computed with emmeans [43]. The emmeans package does not support clmm2. As a result, a cumulative link Mixed Model (clmm) from the ordinal package was used and the output from both models was compared. The results from both models were comparable across all hypotheses, and as such the emmeans package was employed for analysis. When the POA was estimated to be valid, the clmm2 models were retained for use with the predict function, given that the predict function does not support the clmm model. A Proportional Odds Logistic Regression (polr) from the MASS package [44] was used instead of clmm2 when the POA was estimated to be invalid. This model does not support random effects; hence no random effect was included. The brant test [45] was used to test the POA of the polr model. Error rates and standard errors were calculated from assessment scores from the second trial period using Excel to gain insight into the species composition effect within the outsider group according to the location parameters. Overall error rates and standard errors were calculated per species. The following packages were used as complementary tools for plotting, organizing, and analyzing the data: dplyr [46], Hmisc [47], readxl [48], reshape2 [49], tidyr [50] and patchwork [51]. All raw data, scores generated from the analysis and relevant details are presented in the S1 Data.

## Results

Table 2 shows the p-values to assess the statistical significance of the effects tested by comparing the reference factor level to the other factor levels. The location effect is only significant when the plants are outsiders. There is a highly significant difference when comparing Ontario to No Location and Ottawa to No Location (p < 0.001). However, this significance is lost when the plants are established. The difference between Poaceae and Asteraceae plants is very significant (p < 0.01), while the difference between Asteraceae and other plant families is not significant. Additionally, comparing pictures of leaves to pictures of an inflorescence(s) shows a significant difference (p < 0.05), but this is not the case when comparing whole plant pictures to inflorescence pictures. The difference between native and introduced plants is not significant. The effect of tool type is significant when comparing PlantNet to iNaturalist when assessing the effect of plants families (p < 0.05) and the effect of plant distribution status (p < 0.01). However, there is no significant difference between Poaceae plants using PlantNet and Asteraceae plants using iNaturalist, nor between other families using PlantNet. The same lack of significance is observed for native plants using PlantNet compared to introduced plants using iNaturalist. Therefore, the interaction between the family effect or the distribution status effect and the tool type is small and other factors are at play in how either application outperforms the other.

**Table 2:** P-values of the differences between the factor levels and the reference factor level of the tested effects on the taxonomic accuracy assessment obtained through clmm2 modelling.

Effect tested	Reference factor level	Other factor levels	p-Value	
			Established	Outsider
**Location**	No Location	Ontario	0.204	4.240E-50***
		Ottawa	0.964	1.181E-87***
**Family**	Asteraceae	Other	0.387	N/A
		Poaceae	7.748E-03**	N/A
	iNaturalist	Plant Net	1.495E-02*	N/A
	Asteraceae: iNaturalist	Other: Plant Net	0.584	N/A
		Poaceae: Plant Net	0.968	N/A
**Plant parts**	Inflorescence	Leaves	1.394E-02*	N/A
		Whole Plant	0.129	N/A
**Distribution status**	Introduced	Native	0.455	N/A
	iNaturalist	Plant Net	2.361E-04**	N/A
	Introduced: iNaturalist	Native: Plant Net	0.998	N/A

* p ≤ 0.05, ** p ≤ 0.01, *** p ≤ 0.001. N/A: not applicable.

**Fig 2:**
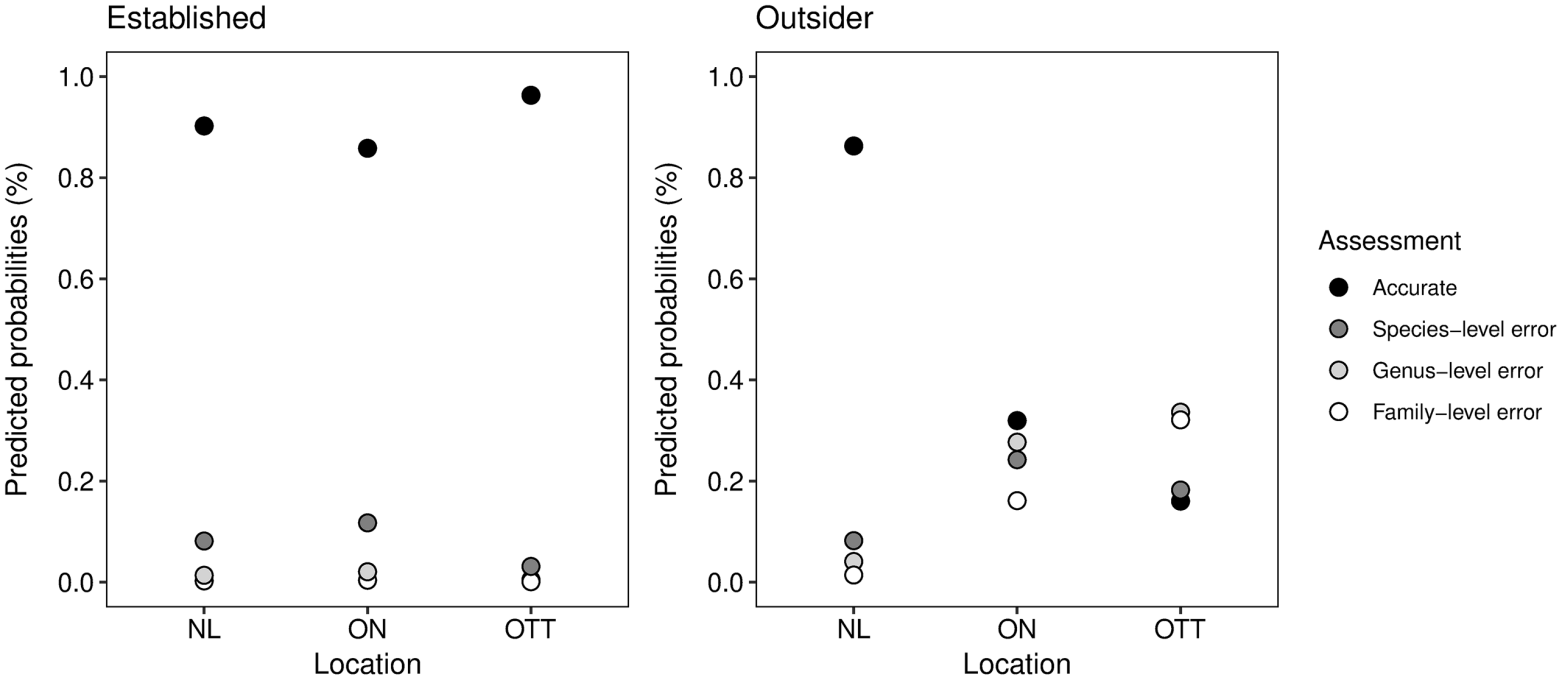
Predicted probabilities of established and outsider plants according to taxonomic accuracy and location using iNaturalist. Location parameters: No Location (NL), Ontario (ON) and Ottawa (OTT). The difference in outsider plants between No Location and Ontario or Ottawa is significant (p < 0.001).

**Fig 3:**
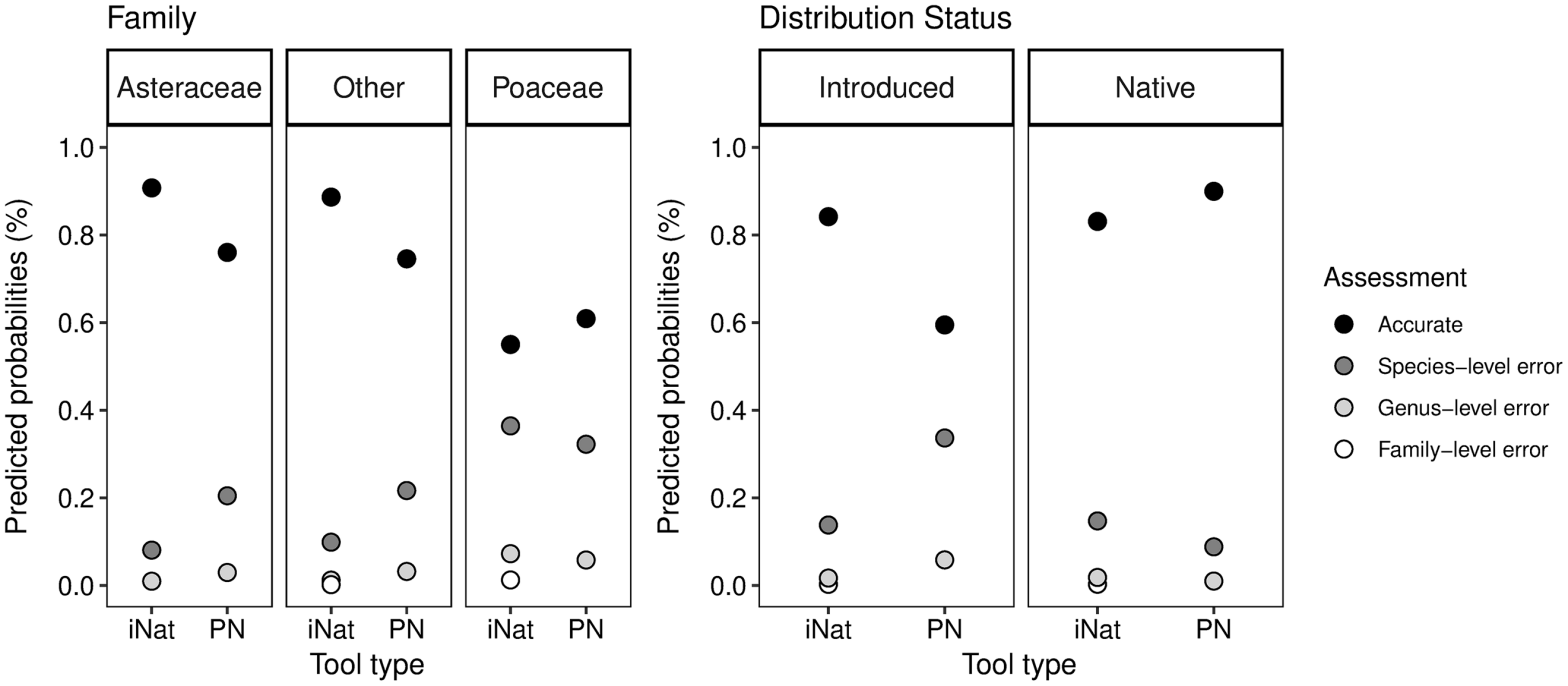
Predicted probabilities of established plants according to taxonomic accuracy, family, status, and tool type interaction. Tool type: iNaturalist (iNat) and Plant Net (PN). The difference between Poaceae and Asteraceae is significant (p < 0.01) and between PlantNet and iNaturalist (p < 0.05 in the family modelling; p < 0.01 in the distribution status modelling).

**Fig 4:**
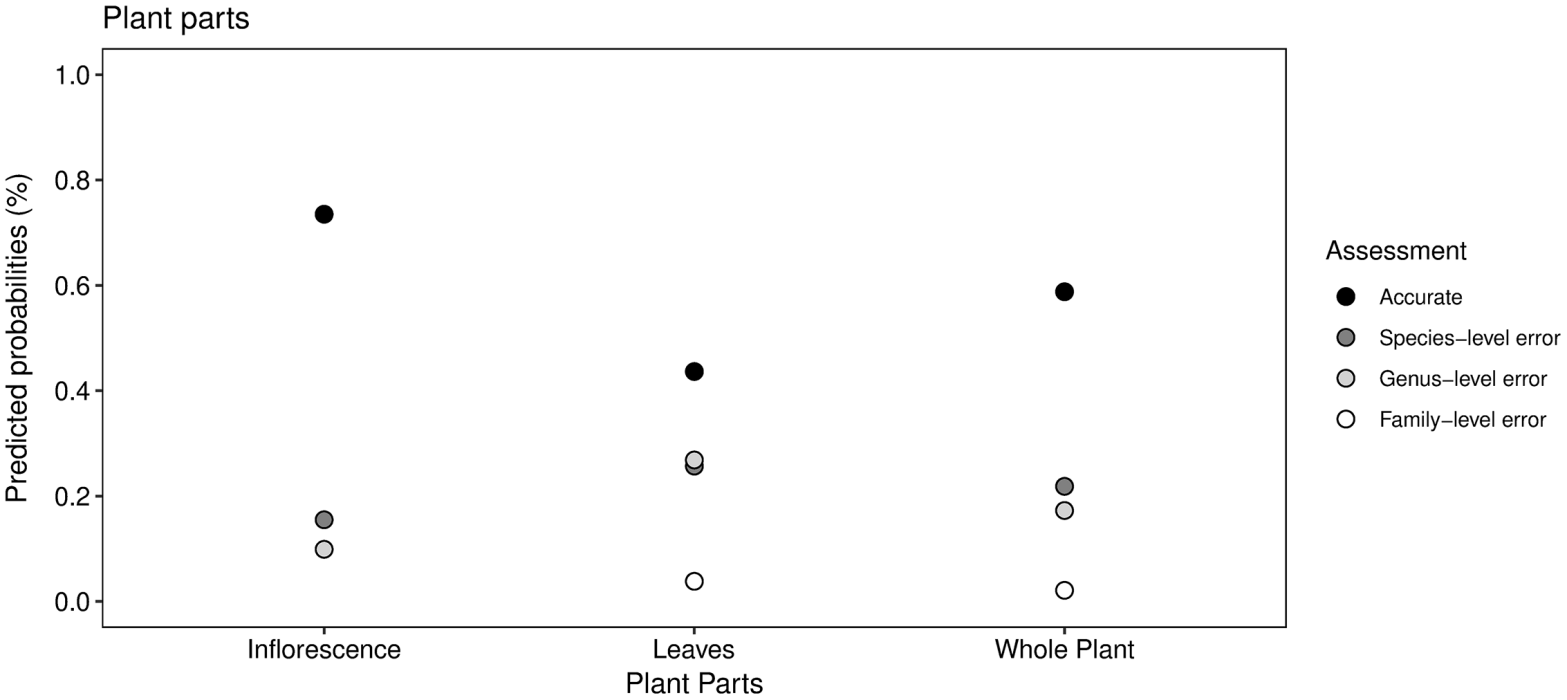
Predicted probabilities of established plants according to taxonomic accuracy and plant parts using iNaturalist. The difference between pictures with only leaves and with only an inflorescence(s) is significant (p < 0.05).

The predicted probabilities for taxonomic accuracy assessment scores based on the effects tested are illustrated in Figs 2-4. The outsider plants exhibit a high predicted probability at the correctly identified level when the input is set to NL (Fig 2). Additionally, when the location is set to Ontario, the predicted percentage is notably greater compared to when it is set to Ottawa. This observation is further emphasized by the error at family level reaching its highest predicted probability when the location is set to Ottawa. In the case of established plants, a high predicted probability at the correctly identified level is noticeable across the different location parameters and remains relatively consistent. Overall, these findings highlight differences in taxonomic accuracy levels between outsider and established plants under varying location conditions and agree with the levels of significance established by the p-values.

Plants in the Poaceae family are less likely to be correctly identified that those in the Asteraceae family or other families (Fig 3). They also have a higher predicted probability of errors at the species level. In terms of tool type, iNaturalist generates more accurate suggestions than PlantNet for plants in the Asteraceae family or other families. In comparison, PlantNet demonstrates a marginally higher predicted probability of accurate identifications for plants in the Poaceae family. The figure also reveals that the taxonomic accuracy of native and introduced plants is largely similar. Both groups demonstrate a high predicted probability of accurate identifications, irrespective of their classification. However, iNaturalist exhibits a higher probability of accurate identifications compared to PlantNet when plants are introduced. Conversely, PlantNet slightly outperforms iNaturalist when plants are native, though the difference is minimal. The predicted probabilities indicate that pictures containing only leaves are less likely to be accurately identified compared to those featuring only inflorescences or whole plants (Fig 4). In addition, pictures with only inflorescences yield higher identification accuracy than those of the whole plant; however, the difference is not statistically significant.

Table 3 presents the error rate (%) and standard error, combining all error levels of the outsider plant species according to the location settings. Most species have similar error rates across the different location settings, except for *E. plantagineum* and *S. elaeagnifolium*. *E. plantagineum* has the highest error rate at 18% when no location is inputted and *S. elaeagnifolium* consistently has the lowest error rate across all location settings. For any given species, the error rate rises as the location precision increases and grasses have the highest error rate in general.

**Table 3:** Error rate and standard error of outsider plants according to location using iNaturalist (n = 402). The errors at every level of the taxonomic accuracy assessment were combined to calculate the overall error rate and standard error.

Location	Results	A. cylindrica*	A. myosuroides*	*E. plantagineum*	E. villosa*	M. vimineum*	*P. montana*	*S. elaeagnifolium*
**No location**	**ER (%)**	13.514	14.000	**18.181**	14.000	17.143	8.696	**7.500**
	**SE (%)**	5.620	3.470	5.815	3.470	6.370	4.154	4.165
**Ontario**	**ER (%)**	70.270	89.000	31.818	62.000	68.571	67.391	**17.500**
	**SE (%)**	7.514	3.129	7.022	4.854	7.847	6.912	6.008
**Ottawa**	**ER (%)**	100.00	100.000	100.000	88.000	100.000	100.000	**67.500**
	**SE (%)**	0.000	0.000	0.000	3.250	0.000	0.000	7.406

ER: error rate, SE: standard error. * Species from the Poaceae family.

At the kingdom-level error, four samples from the outsider group accounted for 0.414% of the samples tested across all location parameters (n = 1209). Two of these are *S. elaeagnifolium* with the location parameter set to OTT, one is *M. vimineum* with the location set to ON, and one is *E. villosa* with the location parameter set to OTT and ON. Only one sample of *P. montana* (Catalog no. 184919384) produced no suggestions across all three location parameters (Table E in S1 Data). The coefficients and intercepts values from the clmm2 and clmm models are comparable (Table A in S1 Data). Results from the emmeans test suggest that the POA is valid for all tested models, except for the location effect with the outsider plant group (Table B in S1 Data). For the location effect, the polr model that was used instead provided similar coefficients and intercepts values to those of the initial clmm2 model (Table A in S1 Data). Furthermore, the brant test confirmed that the POA is valid for the polr model (Table C in S1 Data).

## Discussion

### Location as an input

We assessed if location has a significant effect on the suggestions provided by the computer vision tool and found that there is a strong effect on plants not established in an area (Fig 2, Table 2). We predicted that as the precision of the location input increases, established plants would be identified more accurately, while the misidentification of outsider plants would increase. Our results confirmed only the latter and that the differences between location precision are statistically significant. The established plant group primarily consists of common species that are widespread in and outside Canada (Table D in S1 Data). Common species are often over-represented in the datasets used to train applications. A study by Callaghan et al. [52] found moderate evidence indicating that common bird species were disproportionately represented in iNaturalist. This may explain the lack of difference in identification accuracy between the varying degrees of location preciseness. Furthermore, sampling was conducted in disturbed sites and near roads. Multiple studies have shown that observations are overrepresented in accessible areas, developed areas, managed gardens, or sites near roads or trails [12,53,54]. As a result, computer vision is probably well trained for most of the species in this group. Nonetheless, this result provides direct evidence of a significant bias in iNaturalist, known as spatial bias. This bias causes species that have been observed nearby to be recommended more frequently than those that have not been previously observed in that location. A study conducted by Terry et al. [11] found that geographically constrained identifications tend to be more effective than identifications without such constraints. However, they raised the point that significantly reduced accuracy would result for rarely observed species. Here, we have shown that this is true for species that are scarce in a country where established populations or frequent encounters are lacking. Although we have not tested how sampling bias related to species commonness affects suggestions, we believe this factor likely influences the tool’s performance.

**Fig 5:**
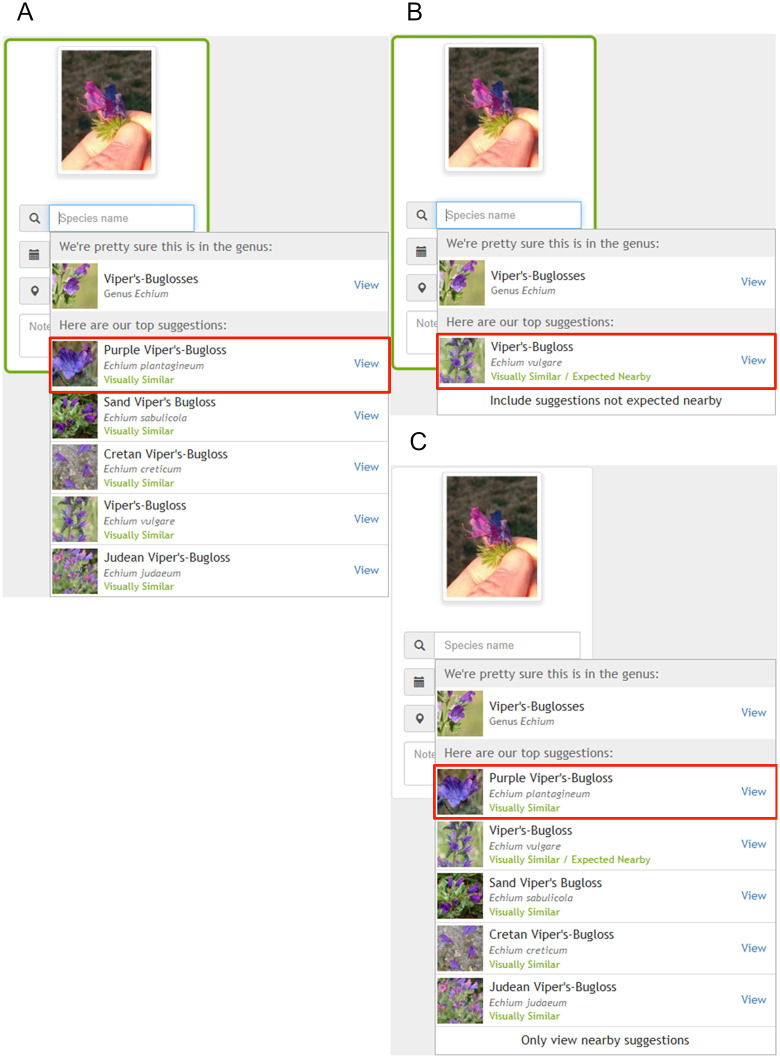
Example of iNaturalist suggestions when location is not used and used as an input. **A)** Includes suggestions returned when location is not inputted. **B)** Includes suggestions returned when Ottawa is inputted as the location. C) includes suggestions returned from selecting “Include suggestions not expected nearby” below in B. iNaturalist image of *Echium plantagineum* was used and we can observe that this species is not among the suggestions until we selected “Include suggestions not expected nearby” when the location is used as an input. Observation 127042112, by brianvanhezewijk [Brian Van Hezewijk], 19/Jul/2022, license is CC BY-NC 4.0. This identification was confirmed by author (AC) as *Echium plantagineum* with a dried collected specimen sent to the CFIA by Brian in 2024.

The spatial bias is raising concerns about potential for missed early detections of invasive species. Since the application’s identification recommendations are strongly influenced by spatial data, the outcome can depend largely on how the user records the observation’s information. iNaturalist recognizes that the Geomodel, which uses location data as an input to return suggestions alongside computer vision, is not perfect and are making improvements to enhance the tool’s accuracy [33]. As such, users that rely on the computer vision tool for identification should enter the location parameter after selecting from the suggested species, as then iNaturalist will only use computer vision to provide suggestions based on visual similarity. If the location is inputted beforehand, the Geomodel will return suggestions with the “Expected Nearby” label. Users should take notice and look for the option to include suggestions not expected nearby. An example demonstrating all three cases, with an image of *Echium plantagineum* from iNaturalist, can be seen in Fig 5. This observation demonstrates how an outsider species might be missed depending on how a user records the observation. It is important to note that the date and location of an observation uploaded to iNaturalist from an iPhone, iPad or android phone should automatically be added depending on the privacy settings or camera GPS capabilities. In contrast, this information is not added automatically when uploading on the web-interface [55]. While this automation is practical, users are encouraged to make decisions on species identification to the best of their knowledge and judgement. Most species suggested after selecting to include suggestions not expected nearby were accurate (AC’s personal observation).

Despite this concern, successful detections remain achievable in iNaturalist, even if they are not the result of AI-assisted identifications. The observation of *E. plantagineum* in Canada by Brian Van Hezewijk illustrates this perfectly (Fig 5). Brian sent dried flowers from his observation to the CFIA, where the identification was confirmed. Subsequently, the CFIA visited the site and discovered that the small population was no longer present, as the area had been paved over. Although no other plants were found, this type of detection allows monitoring agencies to remain vigilant and investigate possible pathways of entry. The site continues to be monitored by the CFIA. In this instance, the user probably did not select a computer vision suggestion because the small icon used for such identifications is absent. Other success stories exist from using iNaturalist to detect new invasions or distributions [56–59]. For example, Fisher et al. [57] detected a new invasion of the invasive lizard species *Aspidoscelis sonorae*, which had previously been misidentified as the native *Aspidoscelis hyperythrus*, by examining photographs labeled as *Aspidoscelis* sp. in iNaturalist. This case underscores the importance of examining genus-level observations, especially for look-alikes, by scanning citizen-science records to monitor IAP.

### Other outcome predictors

#### Plant family.

The identification of Poaceae species is challenging for plant identification tools. The results show that predicted accuracy is just near 60% across the tools (Fig 3). We predicted that applications would underperform for plants from this family due to the small and inconspicuous features of grasses that often require dissection to make identifications. To highlight the difficulty of differentiating species, a notable example from the established plant group is that of confusion between *Echinochloa crus-galli* (L.) P.Beauv. and *Echinochloa muricata* (P.Beauv.) Fernald. These species are distinguished morphologically by the presence or absence of a line of minute hairs at the base of the tip of the upper lemma’s apex [60].This characteristic cannot be captured by a camera alone; it requires the use of a strong lens and dissection. Since most users are unlikely to produce images showing this characteristic, lack of quality training images may lead to unreliable species distinctions within the applications. Pärtel et al. [28] observed a similar trend in plants in the Poaceae family, as well as in other families lacking conspicuous flowers (e.g., Polygonaceae). They noted that the reduced performance may be due to inadequate photography of the flowers’ minute structure or suboptimal perspectives, such as top-down views. Other studies have made the same remark, where diagnostic traits necessary for identification cannot always be captured through photographs [11,26]. Challenges in distinguishing between species that are very similar to one another were also noted in Pärtel et al. [28]. Although there is no significant difference in the results between plants in the Asteraceae family and those from other families in this study, we believe that testing more species from specific families within the other families in this study would likely reveal poorer performance in the applications. For example, species from the Amaranthaceae family that have small flowers and include many dioecious species, may be difficult to identify [61]. In dioecious species of this family, male and female flowers are dimorphic, and if applications are not trained to recognize both forms, errors may occur.

While computer vision may be less effective in identifying small and inconspicuous plants, the lack of accurate identification power could also stem from users under-reporting certain species in the applications, over-reporting false identifications or possibly a combination of all three factors. Several studies have indicated that citizen observers on these applications tend to sample groups with more distinctive morphological features, potentially leading to biased results towards more frequently observed plants. Similar trends have been observed in studies on citizen science data for bird observations, where large-bodied birds were found to be over-represented in sampling by citizens [52]. While size appears to play a significant role in the sampling process by participants, studies have demonstrated that organisms that are more colorful or visually striking are sampled more frequently and are more reliably identified than other organisms in these applications [29]. Furthermore, a separate study of user participation on iNaturalist revealed that 34% of users focused almost exclusively on showy or charismatic insect groups such as butterflies and dragonflies [53]. These findings highlight the biases in the observations made by users on iNaturalist. Since the application relies on user observations to reinforce its computer vision model, these biases can have significant implications on the suggestions made by the model itself. As a result, plants that are larger or more visually striking may be sampled more frequently and thus recommended more frequently as potential identifications. In addition, users confirming misidentifications could lead to lower performance of the applications.

#### Plant parts.

Among the single image categories tested, the results indicate that uploading a single image of leaves provides the least accurate suggestions, as predicted. The difference between uploading a single image of leaves versus uploading an image of either flowers or whole plant proved to be significant even with a small sample size (n = 29). The lack of significant difference between uploading images of only inflorescences compared to uploading images of the plant as a whole may be explained by the presence of an inflorescence in the image. In Hart et al. [62], the performance of the application Seek was significantly higher when a flower was present in the image. Multiple studies have demonstrated that including images of only leaves results in lower performance from computer vision across different applications [28,62,63]. As such, it may be concluded that leaf morphology is not sufficient for accurate identification. Greater accuracy may be obtained using inflorescences, which generally contain more distinctive morphological features. This is usually the case when following the traditional method of plant identification with dichotomous keys. The effect of including multiple images compared to a single image was not tested here. However, we believe that including multiple images would most likely yield more accurate results as some relevant identifying characteristics are not always only present in the reproductive structures, such as the silvery midvein on leaves of *M. vimineum* or its stilt roots [64].

#### Distribution status.

Our findings agree with our prediction that there should not be any significant difference in the identification accuracy between the introduced and native plants from the established plant group. Many introduced plants from our study, such as *Echium vulgare* L. and *Daucus carota* L., have established stable populations in North America and are widely regarded as naturalized [65]. Given that many of these plants were introduced over 200 years ago and are now prevalent, there should not be significant differences between introduced and native plants in the identification outcomes generated by applications because there are ample training images for both. Furthermore, some of the introduced plants included in this study are considered weedy and widespread in anthropogenic areas and disturbed sites, as is the case of *A. retroflexus* L. [66]. As noted earlier, several studies have highlighted biases in observational data linked to the observer’s sampling locations, where observations are overrepresented in accessible and developed areas or near roads [12,53,54]. The selected native plants in this study are common and were collected with the introduced plants. As such, to observe differences within established plants it could be interesting to explore the observer bias, based on the sampling location by selecting plants from various sites.

### Tool type

Based on our findings, we cannot conclusively determine which tool, iNaturalist and PlantNet, provides more accurate identification suggestions. While there is a significant difference between the tools overall, this difference is not statistically significant when considering the interaction of tool type with family and status effect. Although Fig 3 shows slight advantages for each application depending on the plant family or parts, these differences are insufficient to establish which tool is superior for accurate identifications. The small sample size (n = 61) may limit our ability to detect meaningful differences between the tools. As of 2024, PlantNet reports around 7 million users, over 24 million observations and approximately 54,000 species on its statistics page. In comparison, iNaturalist has about 8.2 million users, nearly 87 million plant observations, and about 167,000 species reported on its statistics page or through the Explore query. The greater number of observations and species in iNaturalist suggests it may have an advantage over PlantNet. However, a higher quantity of data does not guarantee better results. Previous research has examined the accuracy of plant species identification across multiple apps, including iNaturalist, Seek by iNaturalist and PlantNet, with many studies showing that PlantNet outperforms iNaturalist or Seek [62,63,67]. While Seek provides suggestions based on data from iNaturalist [68], Campbell et al. [63] found that iNaturalist outperformed Seek, which was among the lowest performing apps. The authors did not provide an explanation for this discrepancy, though we would expect both tools to provide similar suggestions since they are trained on the same data [68]. In this same study, it is important to note that accuracy was assessed based on the “pretty sure” taxon identification, which appears above the list of “top suggestions” in iNaturalist’s computer vision window. However, accuracy should be evaluated on the species listed in the “top suggestions” themselves, as the “pretty sure” result only provides a broad, confident identification at the family or genus level. Although our study’s sample size for this test is small and testing was conducted via the web interfaces rather than smartphones, we believe we have developed a fair and robust method for assessing the performance of both iNaturalist and PlantNet, supported by the use of CLMM modeling.

### Simultaneous effects at play

While we have highlighted a significant location effect on the outsider plant group, we must also take into consideration the species composition within this group and its impact on iNaturalist’s suggestions. Four of the species belong to the Poaceae family, which, as we noted with the family effect, may influence the results. As observed in Table 3, the computer vision underperforms the most for species in this family among the outsider group, as fine morphological differences among species characteristics are difficult to capture in photographs. Similarly, *E. plantagineum*, a species from the outsider group*,* is distinguished from its lookalike *E. vulgare* by the number of stamens protruding beyond the corolla [65]. For accurate identification, this feature must be clearly visible in photographs, which can be difficult to capture due to variations in camera angles and lighting. As outlined in Terry et al. [11], picture quality is crucial for the computer vision models to pick up morphological features, and a lack of features may lead the model to rely more heavily on spatiotemporal data. The pictures selected of *E. plantagineum* for testing on iNaturalist were all verified for this characteristic, confirming the identification. However, the application may lack the training necessary to differentiate between the two species and may rely more on the location effect. Our results suggest this is likely true as the more the location precision increases, the more the error rate increases. To further test this hypothesis, pictures of confirmed *E. vulgare* with visible stamens and without could be used along with pictures of *E. plantagineum* in a similar way, with no location inputted.

Additionally, most pictures of *P. montana* used in testing iNaturalist primarily featured leaves. We noticed that many observations of *P. montana* on iNaturalist lacked inflorescences or full plant images, likely due to the vine-like nature of the plant. As noted, pictures only including leaves often result in less accurate identifications. The nightshade species, *S. elaeagnifolium*, is visually striking due to its silver leaves and stems and large purple flowers with yellow anthers, which should yield better identification results. As shown in Table 3, the identification of this species is consistently slightly better than that of the other species, regardless of the location setting within this group. Nevertheless, the location effect still influences the results for this more conspicuous species. Despite the possibility of other effects interacting with the location effect or having their own influence, we demonstrated that when plants are not established in an area, it does affect the suggestions made by iNaturalist.

### Limitations, biases and assumptions

Our study is based on several assumptions and limitations. An important underlying assumption is that computer vision is uniformly trained across all species included. While we acknowledged that differences may exist within the species composition of the established and outsider groups, our findings indicate that the location effect remains significant. We also assumed that the level of training for the computer vision is consistent across all taxonomic accuracy assessment testing periods and that picture quality is comparable among samples. However, with iNaturalist releasing a new trained computer vision model each month [33], it becomes challenging to test a large number of samples and different effects. We also assumed that using pre-existing pictures from iNaturalist observations, possibly used to train the model, does not interfere with the identification suggestions made. Additionally, we assumed that the suggestions provided by the tool’s web interface are like those from smartphone apps. In terms of limitations, testing a larger number of established plants is necessary to identify which plant identification tool delivers the greatest accuracy. Despite the small sample size, our findings on family effects and plant parts are consistent with previous research. Furthermore, we occasionally used objects or backgrounds to help bring the plant in focus when taking pictures. This approach was employed to enhance recognition and get a suggestion, especially for fine grass spikelets. We believe that users often utilize similar aids to achieve better outcomes (e.g., hand). Finally, this study addressed the potential impact of three biases on the identification suggestions made by applications, see Table 4. Other biases, related to sampling bias, are certainly influencing the observations reported on these applications.

**Table 4:** Summary of addressed biases and their effect on the identification suggestions made by applications.

Biases	Effect
**Spatial**	Species expected nearby will be more frequently suggested as they are over-represented in accessible locations, developed areas, etc. in the dataset.
**Showy**	Larger, distinctive, and charismatic species will be more frequently suggested as they are well represented in the dataset.
**Common**	Widespread, abundant, or frequent species may be more frequently suggested as they are well represented in the dataset.

The sample sizes for each effect tested were 463, 61, 61, and 29 for location, family, distribution status and plant parts, respectively, with tool type included as an interaction for some analyses (Table 1). The smaller sample size associated with some effects were not considered a cause of concern. The taxonomic accuracy scores used in this study are ordinal in nature and therefore comparable to Likert-scale data. Previous analyses comparing parametric and non-parametric testing for such ordinal data have shown that both methods perform comparably when sample size exceed fifteen, with parametric tests generally offering greater discriminatory power among groups [69]. Moreover, assumptions underlying parametric tests do not involve sample size [70], and smaller samples sizes generally reduce statistical power rather than inflate error rates. All sample sizes used here were well above the fifteen-sample threshold. While smaller sample sizes can lead to wider confidence intervals due to greater standard errors, this reflects reduced precision rather than bias and does not comprise the validity of the inferences drawn. Additionally, the assumptions of the cumulative link mixed models applied in this study were tested and found to be satisfied. Finally, the results obtained were consistent and logical, further supporting the robustness of the statistical approach employed (Table 2).

## Conclusion

To the best of our knowledge, this is the first study to investigate the impact of location as an input on suggestions generated by computer vision, with significant implications for detecting new invasive plant species. Our innovative use of cumulative link mixed models to analyze ordinal data in AI-based identification applications not only accounts for the inherent ordering of the data but also accommodates random effects, enhancing the interpretability and significance of our findings. Given the larger sample size in the outsider group, we infer that the influence of location as an input can led to the misidentification of IAP. Fortunately, iNaturalist is a community-driven platform where users can make identifications and verify findings collaboratively. Federal Programs, such as the “Regulated Pests of Canada” under the *Plant Protection Act*, effectively utilize iNaturalist to monitor potential IAP detections, including those from the outsider group in this study. We urge those involved in such monitoring programs to conduct genus-level verifications or verify look-alikes to ensure that novel detections are not overlooked. Despite potential biases in citizen science data, these platforms are invaluable due to their extensive data, numerous participants, and wide geographic coverage. Future monitoring efforts should prioritize the development of tools to aggregate detections from various applications to enhance surveillance. Additionally, further exploration of the impact of location in ecological monitoring is essential, especially in the context of climate range shifts affecting both invasive species and native species that may emerge in new habitats.

## Supporting information

S1 Data R code output results, raw data, calculations of errors rates and standard errors. (XLSX)

S1 Code R guidelines and code. (DOCX)
