# Correction: Investigating key drivers influencing AI-based detection and identification of plants

**DOI:** 10.1371/journal.pone.0346524

**Published:** 2026-04-02

**Authors:** Andréanne Charron, Adèle Julien, Joseph R. Stinziano, Marie-Claude Gagnon

The ORCID iD is missing for the third author. Please see the authors’ ORCID iD here:

Author Joseph R. Stinziano’s ORCID iD is: 0000-0002-7628-4201 (https://orcid.org/0000-0002-7628-4201).

In the Author Contributions, Joseph R. Stinziano (JS) should not be listed as one of the persons who wrote the original draft paper.

The following information is missing from the Copyright statement: © His Majesty the King in Right of Canada.
