# Supplementary material for: Investigating key drivers influencing AI-based detection and identification of plants
Source: PLoS One. 2026 Mar 2;21(3):e0342712. doi: 10.1371/journal.pone.0342712 (PMC12952603; doi:10.1371/journal.pone.0342712)
Supplement: S1 Code — (DOCX) [file pone.0342712.s002.docx]

**R guidelines and code**

The following guideline provides the necessary steps to use the code and analyze data with cumulative link mixed models. The code used to analyse the dataset for the location predictor in the established plants is often presented as an example. The integral coding for all predictors can be found at the end of the document. The statistical language R, version R 4.3.2 was used to build and run the analysis. The sections are as follow:

[Variables](#_Variables)
[Preliminary steps](#_Preliminary_steps)
[Running clmm2](#_Running_clmm2)
[Calculating p-values](#_Calculating_p-values)
[Calculating predicted values](#_Calculate_predicted_values)
[Proportional odd assumption testing](#_Proportional_odd_assumption)
[Plotting](#_Plotting)
[Code](#_Script)

# **Variables**

**Dependant variable (ordinal factor): assessment**

| Assessment | correctly identified (0), species-level error (1), genus-level error (2), and family-level error (3) |
| --- | --- |

| **0** | **1** | **2** | **3** | y* |
| --- | --- | --- | --- | --- |

$$\boldsymbol{\theta}\boldsymbol{\theta\theta}$$

**y*** = Latent variable

$\boldsymbol{\theta}$ = Intercepts (3 total), corresponds to the boundary values between the different categories.

**Independent variable: predictors**

| Location | Ottawa (OTT), Ontario (ON), No location (NL) |
| --- | --- |
| Family | Asteraceae, Poaceae, Other |
| Plant parts | Whole plant, Leaves, Flowers/Inflorescence |
| Status | Native or Introduced |
| Tool Type | iNaturalist (iNat) or PlantNet (PN) |

# **Preliminary steps**

**Setting the working directory**

The first step is to set the working directory, which is the default folder on your computer where R looks for files to read (e.g. dataset) and where it saves output files (e.g. plots). In R, *setwd()* is used to set the working directory :

| setwd("insert file path") |
| --- |

*To verify the current file path where the working directory is set, the function *getwd*() will return the file path.

**Loading packages**

Next, multiple packages need to be installed and loaded to use the different functions and arguments to run the script. Only MASS is a built-in package in R, the others will need to be installed before loading. To install, use *install.package(c(…)*. To load packages, you must type *library()*. This will make the package’s functions available in your current R session. All packages will need to be loaded each time you restart R, this includes packages like MASS that are built-in.

The following packages are to be installed and loaded:

| library(brant)  library(dplyr)  library(emmeans)  library(foreign)  library(ggplot2)  library(Hmisc)  library(ordinal)  library(readxl)  library(reshape2)  library(tidyr)  library(MASS)  library(patchwork) |
| --- |

**Importing the dataset**

To import the datasets from Excel files (.xls and .xlsx) into R as a data frame *read_excel* is used. It’s important to save the dataset under the same default folder used for the working directory. The name of the dataset Excel file is required. The operator *<-* is used to assign a variable to the dataset. The variable will hold the dataset and will enable the use of the dataset in further analysis. If the dataset contains multiple sheets, you may specify from which sheet to import from, as follow:

*Insert name for variable <- read_excel(“insert excel file name”, sheet = “insert sheet name”)*

In this study, sheets were used to organize the results obtained for the established plants, the outsider plants and the plant parts.

| Data_Established_long_format <- read_excel("GB AI Project - Data long format.xlsx", sheet = "Established") |
| --- |

| RStudio source panel output |
| --- |
| **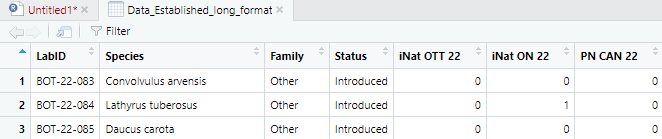** |

**Reorganizing a dataset to long format**

Before changing the dataset into long format, it can be helpful to view the columns in the dataset, the function *colnames*() was used.

| colnames(Data_Established_long_format) |
| --- |

The function *pivot_longer*() reshapes the data. It takes multiple columns and puts them into new columns. *cols = c* (`insert column name`) selects the specific columns you want to pivot. *names_to* *= “insert new column name”* tells R where to store the column names from the wide-format data when you reshape it into long format. The *values_to = “insert new column name”* tells R the name of the new column that will contain the data values from the columns you pivot.

Instead of having many wide columns (one per survey/location/year) with each their data values, the following columns are created: “names” (e.g. iNat ON 23) and their data values under “assessment”.

Here the variable name was modified to “Data_Established” to store the modified dataset in R as separate to the original “Data_Established_long_format”.

| Data_Established <- pivot_longer(Data_Established_long_format, cols = c(`iNat OTT 22`,`iNat ON 22`,`PN CAN 22`,`PN NL 22`,`iNat OTT 23`,`iNat ON 23`,`iNat NL 23`,`PN CAN 23`,`PN NL 23`), names_to = “names”, values_to = “assessment”) |
| --- |

| RStudio source panel output |
| --- |
| 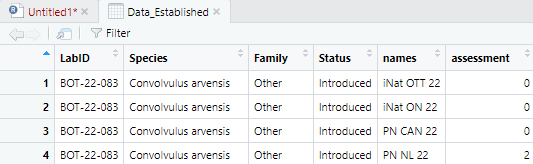 |

Next, the function *separate* () splits one column into multiple columns based on a separator. The argument *col = “insert column name”* selects the specific column to split and the argument *into = c (“new columns names to assign”)* will create a new column from the selected column. The separator *sep = " "* splits wherever there is a space.

Here, this function was used to split the column named as “names” from the Data_Established dataset into three new columns: tooltype, location, year.

| Data_Established_ToolLocationTime <- separate (Data_Established, col = "names", into = c("tooltype", "location", "year"), sep = " ") |
| --- |

| RStudio source panel output |
| --- |
| **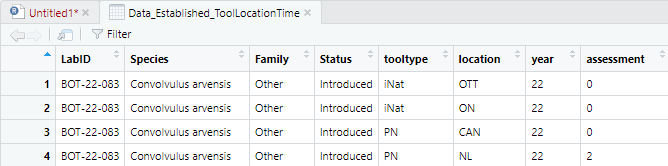** |

**Visualization of the distribution**

To visualize the distribution of assessment values across different years, tool types, and locations, the following script was used. This is useful for quick comparison to see how assessments vary. The function *group_by ()* is only necessary if summaries are added later as *ggplot ()* can handle grouping.

| Data_Established_ToolLocationTime\|> group_by(tooltype,location,year)\|>ggplot(aes(x = assessment, fill = year)) + geom_histogram() + facet_wrap(location ~ tooltype) |
| --- |

**Converting a column into a categorical variable**

To convert a column into a categorical variable (ordered factor), *factor (dataset frame name$column name, levels = c(0:3))* is used. *levels = c(0:3)* defines the possible values in order 0, 1, 2, 3. *Labels = c(" ")* sets the labels for those levels as character labels. *ordered = TRUE* makes it an ordered factor (i.e., 0 < 1 < 2 < 3), which means R will treat it as having a natural order.

This step is essential as the variable assessment corresponds to ordered categorical outcomes: correctly identified (0), species-level error (1), genus-level error (2), and family-level error (3).

| Data_Established_ToolLocationTime$assessment <- factor(Data_Established_ToolLocationTime$assessment, levels = c(0:3), labels = c("0","1","2","3"), ordered = TRUE) |
| --- |

**Saving filtered subsets into a new data frame**

To analyze the independent variables (predictors), new datasets with filtered subsets where created. For comparing location effects on established and outsider plants, the dataset “Data_Established_ToolLocationTime” was restricted to 2023 iNaturalist records, since outsider plants data were only used under these conditions, as outlined in the paper.

Additionnaly, *subset(dataset frame name, select = -Status)* was used to remove the column Status and the result was reassigned to “Data_Established_23iNat”. as.factor (data frame name$column name) was used to convert the LabID or the CatalogID column into a factor variable (categorical) instead of a numeric character as seen below. This tells R to treat LabID or CatalogID as a factor rather than a number.

Since only data from 2023 and using iNaturalist were used for the Outsider plants, data with NA needed to be dropped. *Drop_na* removes any rows that contain missing values (NA) from the data frame, the *<-* saves the cleaned version back into the same object, overwriting it.

| Data_Established_23iNat <- Data_Established_ToolLocationTime[Data_Established_ToolLocationTime$year == "23" & Data_Established_ToolLocationTime$tooltype == "iNat",] |
| --- |
| Data_Established_23iNat <- subset(Data_Established_23iNat, select = -Status) |
| Data_Established_23iNat$LabID <- as.factor (Data_Established_23iNat$LabID) |
| Data_Established_23 <- Data_Established_ToolLocationTime[Data_Established_ToolLocationTime$year == "23",] |
| Data_Outsider_Location <- drop_na (Data_Outsider_Location) |
| Data_Established_23$LabID <- as.factor(Data_Established_23$LabID) |
| Data_Outsider_Location$CatalogID <- as.factor(Data_Outsider_Location$CatalogID) |

| RStudio source panel output |
| --- |
| **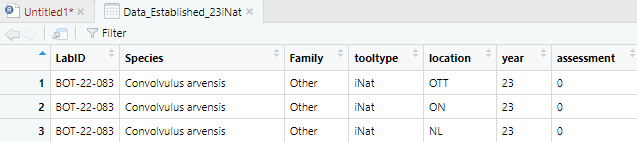** |
| **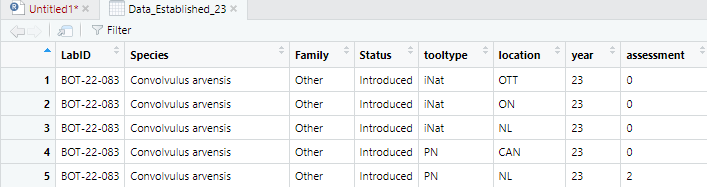** |

**Summaries in R**

To get a quick look at the data and check the distribution of values or factor levels, *summary(…)* will return if it’s numeric, basic statistics (Min, 1^st^ Quartile, etc.), if it’s a factor and a frequency table which tells how many observations fall into each category.

| summary(Data_Established$assessment) |
| --- |

# **Running clmm2**

The Cumulative Link Mixed Model version 2 (clmm2) from the ordinal package, fitted with the adaptive Gauss-Hermite quadrature approximation, was used to evaluate the taxonomic accuracy score, the outcome variable “assessment”. The Gauss-Hermite quadrature approximation was run with 7 quadrature points.

*clmm2()* fits a cumulative link mixed model (CLMM), so an ordinal logistic regression, with random effects. *Outcome variable ~ predictor variable* tells the model to predict the ordinal outcome assessment based on the predictor variable selected: location, family, plant parts and status. The interaction between the predictor variables, plant family and distribution status, with the tool type (iNaturalist and PlantNet) were included with *.

*Random = random effect* tells R to include a random effect, here LabID or CatalogID. This accounts for repeated measures or clustering. *Hess = True* requests computation of the Hessian matrix, needed for reliable standard errors and inference. *nAGQ = 7* sets the number of adaptive Gauss-Hermite quadrature points, which controls how accurately the random effects likelihood is approximated.

| Model_Hypothesis_Location_Established <- clmm2 (assessment ~ location, random = LabID, data=Data_Established_23iNat, Hess = TRUE, nAGQ =7) |
| --- |
| Model_Hypothesis_Location_Outsider <- clmm2 (assessment ~ location, random = CatalogID, data = Data_Outsider_Location, Hess = TRUE, nAGQ=7) |
| Model_Hypothesis_Family_Established <- clmm2 (assessment ~ Family * tooltype, random = LabID, data=Data_Established_23, Hess = TRUE, nAGQ =7) |
| Model_Hypothesis_Plant_Parts_Established <- clmm2 (assessment ~ plantparts, random = LabID, data=Data_Plant_Parts_Established, Hess = TRUE, nAGQ=7) |
| Model_Hypothesis_Status_Established <- clmm2 (assessment ~ Status * tooltype, random = LabID, data=Data_Established_23, Hess = TRUE, nAGQ =7) |

*summary()* is used to look at the results of the model fitted. It shows what was fitted, the link function (logit usually), the thresholds (cutpoints that separate categories of the ordinal outcome), the estimated coefficients, their standard errors, z-values and p-values of the fixed effect (e.g. location), the random effects’ variance estimate(s) for the random intercept(s) the model fit statistics (log-likelihood, AIC, etc.).

| RStudio console panel output |
| --- |
| 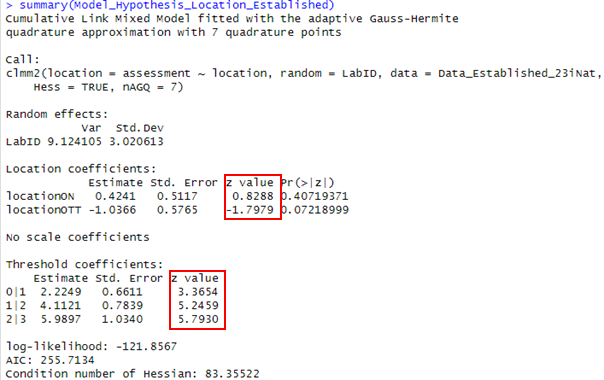 |

The values of the location coefficients in the red squares tells us whether the effect of each location differs significantly from the reference location (No location). 0.8288 is close to 0 and -1.7979 is farther from 0, but still below the typical cutoff of $\pm$1.96 (for p $<$ 0.05). There is no evidence that location ON (Ontario) is different from No location in terms of its effect on assessment. Location OTT (Ottawa) might differ slightly from No location, but the evidence is weak.

The thresholds define the “boundaries” between categories of the ordinal outcome. Threshold z values (3.37, 5.24, 5.79) are well above 1.96. This means they are estimated with high precision, so significantly different from 0. The model is confident about where the category cutoffs lie between correctly identified (0), species-level error (1), genus-level error (2), and family-level error (3).

When interactions are included in the models (e.g. family * tooltype or status * tooltype), the results of the model fitted appear as such:

| RStudio console panel output |
| --- |
| 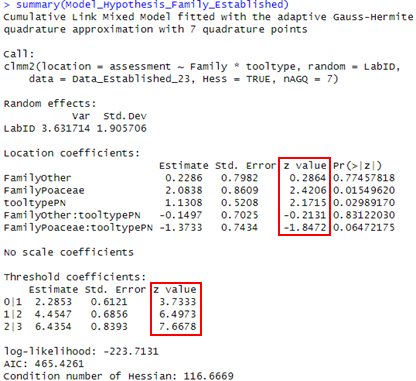 |

The model estimates the effect of family when tool type is at its reference level (iNaturalist) and the effect of tool type when family is at its reference level (Asteraceae), and the interaction (familyX:tooltypeY) which tells you how much the effect of tool type changes when family is not a reference level but a specific category. The interaction is the adjustment to the reference level effect when both predictors take those levels together.

The z-value -0.2131 for familyother:tooltypePN is very close to 0. This means the interaction effect of being in family = other and tooltype = PN is basically non-existent. The z-value -1.8472 still below the cutoff $\pm$1.96 suggests there might be an interaction effect of being in family = Poaceae and tooltype = PN, but the evidence is weak. The z-values show no convincing evidence that PlantNet’s effect changes depending on the plant family.

# **Calculating p-values**

Pr(> | z |) values generated in summaries above correspond to p-values associated with the z-value for a coefficient. Clmm2 does not always show p-values for fixed effects. p-values for mixed models are a bit tricky, it’s better to calculate them manually to exclude threshold rows, control one-tailed vs two-tailed p-values and create a unified table.

*pnorm(…, lower.tail = FALSE)* converts z-values into one-sided p-values. *Coef(summary(…))* extracts the coefficients table from the model. *[,"z value"]* gets the z-values for each coefficient. *[-1:-3]* removes the first three rows, which usually correspond to the thresholds. p-values are calculated for predictors and not thresholds typically.

| pnorm(coef(summary(Model_Hypothesis_Location_Established))[,"z value"], lower.tail = FALSE)[-1:-3] |
| --- |

To organize the calculated p-values, *coef(summary(…))[, c(1,3)]* tells R to keep only column 1 (estimate) and column 3 (z-value) from the coefficients table. *cbind(…)* tells R to combine the estimates, z-values and calculated p-values into one table. Finally, *colnames(…)[3] <- "PR > Z || PR < Z"* renames the third column (p-values) for clarity.

| coef(summary(Model_Hypothesis_Location_Established))[, c(1,3)] |
| --- |
| statsTable_Hypothesis_Location_Established <- cbind(coef(summary(Model_Hypothesis_Location_Established))[, c(1,3)],pnorm(coef (summary(Model_Hypothesis_Location_Established))[,"z value"], lower.tail = FALSE)) |
| colnames(statsTable_Hypothesis_Location_Established)[3] <- "PR > Z \|\| PR < Z" |

| RStudio source panel output |
| --- |
| 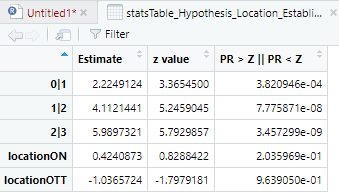 |

# **Calculating predicted values**

To ease the interpretation of clmm2 modelling, predicted values turn coefficients into probabilities which are easier to understand. In addition, it makes it easier as well for visualization and reporting.

*Predict(…, newdata=…)* calculates the predicted values of assessment for each row in the dataset. *cbind(…)* adds the predicted values as a new column (pred) to the original dataset. To keep the original dataset intact, a new dataset is saved as “p_Location_Established”. *as.factor(dataset name$assessment)* converts the assessment column to a factor, so it’s treated as categorical (not numeric) in further analysis or plots.

| p_Location_Established <- cbind(Data_Established_23iNat, pred = predict (Model_Hypothesis_Location_Established, newdata = Data_Established_23iNat)) |
| --- |
| p_Location_Established$assessment <- as.factor(p_Location_Established$assessment) |

| RStudio source panel output |
| --- |
| 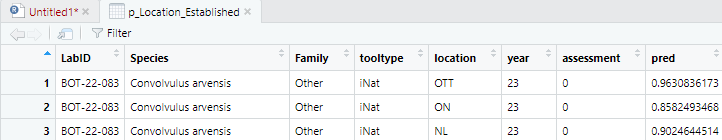 |

# **Proportional odd assumption testing**

CLMMs assume the outcome is ordinal (e.g. correctly identified, species-level error, genus-level error, and family-level error). The proportional odd assumption (POA) states that the effect of each predictor (e.g. location, tooltype) is the same across all thresholds of the outcome. In other words, the relationship between the lowest versus all higher categories of the response variable (assessment) are the same as those that describe the relationship between the next lowest category and all higher categories. The relationship between each pair of outcome groups is equal. If the assumption holds, the model is valid and a single coefficient per predictor can be interpreted for all levels of the outcome.

If the assumption is violated, the effect of the predictor is different for each threshold, and a simple CLMM is not appropriate. Not testing this assumption can lead to misleading coefficients and underestimating or overestimating effects. The assumption can be relaxed when a predictor affects some outcomes thresholds differently from others. This helps make the model more flexible and realistic while still using ordinal structure.

**emmeans()**

clmm2 models doesn’t have a built-in function like brant() for polr models to test the POA. Here, emmeans() was used to see if the effect of a predictor is very different across categories (thresholds). Emmeans basically calculates predicted probabilities for each threshold level, averaging over the other threshold levels in the model. So, it does not formally test the POA, but it can suggest if there’s a violation of the POA. However, clmm2 does not support emmeans always. In such case, a clmm model is required. A clmm model was fit for each predictor and the summary() was used to compared t-values to the z-values generated with the clmm2 models. The results were comparable (Table A in S1 Data) and for this reason emmeans was used with clmm models to see if the POA might be violated.

| Clmm_Hypothesis_Location_Established <- clmm(assessment ~ location + (1\|LabID), data= Data_Established_23iNat, Hess = TRUE, nAGQ =7) |
| --- |
| summary(Clmm_Hypothesis_Location_Established) |
| emmeans_Location_Established <- emmeans (Clmm_Hypothesis_Location_Established, list(pairwise~location)) |
| summary(emmeans_Location_Established) |

| RStudio console panel output |
| --- |
| 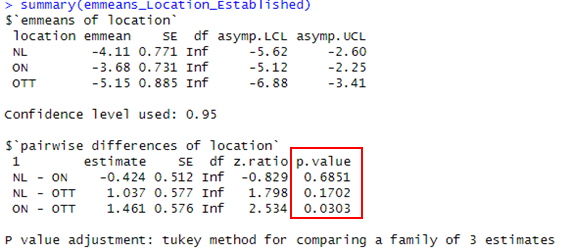 |
| 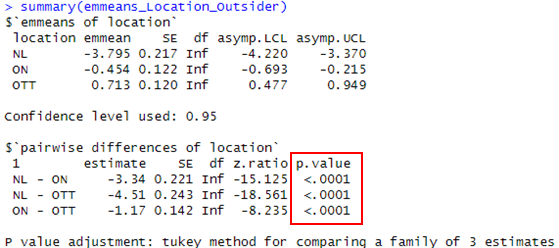 |

The p-values from emmeans are not significant (p > 0.01) for the clmm model used to test location as a predictor in established plants. This suggests the POA is true. However, the p-values are significant (p ≤ 0.001) for the clmm model used to test location as a predictor in outsider plants. This suggests the POA is violated. As such, clmm2 or clmm are not appropriate models to analyze the effect of location on outsider plants.

**brant()**

To accommodate for this violation, the proportional odds logistic regression (polr) from the MASS package is used to analyze the effect of location in outsider plants and to make comparisons with the established plants. This model assumes the proportional odds but does not handle random effects. The following steps, which are almost similar to those used earlier, were carried out to fit the polr model, calculate p-values, calculate predicted probabilities, and plotting:

| Model_Hypothesis_Location_Outsider_polr <- polr(assessment ~ location, data = Data_Outsider_Location, Hess = TRUE) |
| --- |
| summary(Model_Hypothesis_Location_Outsider_polr) |
| pnorm(coef(summary(Model_Hypothesis_Location_Outsider_polr))[,"t value"], lower.tail = FALSE)[-1:-3] |
| coef(summary(Model_Hypothesis_Location_Outsider_polr))[, c(1,3)] |
| statsTable_Hypothesis_Location_Outsider_polr <- cbind(coef(summary(Model_Hypothesis_Location_Outsider_polr))[, c(1,3)],pnorm(coef (summary(Model_Hypothesis_Location_Outsider_polr))[,"t value"], lower.tail = FALSE)) |
| colnames(statsTable_Hypothesis_Location_Outsider_polr)[3] <- "PR > Z \|\| PR < Z" |
| p_Location_Outsider_polr <- cbind(Data_Outsider_Location, predict(Model_Hypothesis_Location_Outsider_polr, type = "probs")) |
| p_Location_Outsider_polr <- melt(p_Location_Outsider_polr, id.vars = c("CatalogID", "Species", "tooltype", "location", "year", "assessment"), value.name = "Probability") |
| plot2 <- ggplot(p_Location_Outsider_polr, aes(x = location, y = Probability , fill = variable)) + geom_point(shape = 21, size =3, color = "black") + scale_fill_manual(name = "Assessment", values = c("0"= "#000000", "1" = "#808080", "2" = "#D3D3D3", "3" = "#FFFFFF"), labels = c("0"= "Accurate", "1"= "Species-level error", "2"= "Genus-level error", "3"= "Family-level error")) + labs(title = "Outsider", x = "Location", y = "Predicted probabilities (%)") + scale_y_continuous(limits=c(0.00, 1.00), breaks = seq (0.00, 1.00, by = 0.20)) + theme(axis.text.x = element_text(size = 11, color = "black"), axis.text.y = element_text(size = 11, color = "black"), axis.title.x = element_text(size = 12, color = "black"), axis.title.y = element_text(size = 12, color = "black", margin = margin(r =10)), panel.background = element_rect(fill = "white"), panel.border = element_rect(color = "black", fill = NA), panel.grid.major = element_blank(), panel.grid.minor = element_blank()) |

The polr model provided similar coefficients and intercepts values to those of the initial clmm2 model for testing the location effect (Table A in S1 Data).

*brant()* tests the POA for ordinal logistic regression models, like *polr()* from MASS.

| brant(Model_Hypothesis_Location_Outsider_polr) |
| --- |

| RStudio console panel output |
| --- |
| 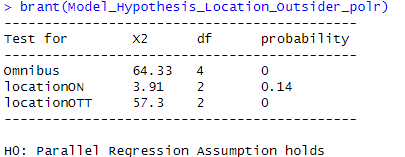 |

p-values are not significant (p > 0.05) and as such, the assumption holds. The polr model is appropriate to test the location effect on the outsider plants.

All emmeans and brant test results are in Table B and Table C in S1 Data.

# **Plotting**

To create plots with observed data and predicted values *ggplot(…)* is used. *aes(x = location, y = pred, fill = assessment* tells R the x axis will be the location predictor, the y axis will be the predicted values from the pred column and to color each point based on the type of assessment category.

*labs(title = " ", x = " ", y = " ")* sets the plot title and the x-axis and y-axis labels. *geom_point* customizes the points in the plot (shape, size, and color). *scale_fill_manual(name = " ", values = c(" " = " ", …)* assigns specific colors to each assessment category and assigns labels for the legend. *theme( )* controls the appearance of axes, labels, background, and grid. *scale_y_continuous* limits y-axis from 0 to 1 (0% to 100% probability) and adds tick marks every 0.2 (20%).

| plot1 <- ggplot(p_Location_Established, aes(x = location, y = pred, fill = assessment)) + geom_point(shape = 21, size =3, color = "black") + scale_fill_manual(name = "Assessment", values = c("0"= "#000000", "1" = "#808080", "2" = "#D3D3D3", "3" = "#FFFFFF"), labels = c("0"= "Accurate", "1"= "Species-level error", "2"= "Genus-level error", "3"= "Family-level error")) + labs(title = "Established", x = "Location", y = "Predicted probabilities (%)") + theme(axis.text.x = element_text(size = 11, color = "black"), axis.text.y = element_text(size = 11, color = "black"), axis.title.x = element_text(size = 12, color = "black"), axis.title.y = element_text(size = 12, color = "black", margin = margin(r = 10)), panel.background = element_rect(fill = "white"), panel.border = element_rect(color = "black", fill = NA), panel.grid.major = element_blank(), panel.grid.minor = element_blank()) + scale_y_continuous(limits=c(0.00, 1.00), breaks = seq (0.00, 1.00, by = 0.20)) |
| --- |

To combine plots together, the following was inputted in R:

| plot_Location_Combined <- plot1 + plot2 + plot_layout(ncol = 2, guides = "collect") |
| --- |
| plot_Family_Status_Combined <- plot3 + plot5 + plot_layout(ncol = 2, guides = "collect") |

By default, plots would be stacked vertically. *ncol = 2* arranges the plots in 2 columns side by side. *guides = "collect"* combines legends from both plots into one shared legend.

# **Code**

**### Save working directory for GB-AI Project**

setwd("N:/OPL-LPO/Genotyping-Botany/Diagnostics/3. Botany/Artificial Intelligence Research Projects/GB Project/R Analysis (Stats)")

**### Packages to install and load**

library(brant)

library(dplyr)

library(emmeans)

library(foreign)

library(ggplot2)

library(Hmisc)

library(ordinal)

library(readxl)

library(reshape2)

library(tidyr)

library(MASS)

library(patchwork)

**### Predictor 1a - Established Plants**

**# Data organization**

Data_Established_long_format <- read_excel("GB AI Project - Data long format.xlsx", sheet = "Established")

colnames(Data_Established_long_format)

Data_Established <- pivot_longer(Data_Established_long_format, cols = c(`iNat OTT 22`,`iNat ON 22`,`PN CAN 22`,`PN NL 22`,`iNat OTT 23`,`iNat ON 23`,`iNat NL 23`,`PN CAN 23`,`PN NL 23`), names_to = "names", values_to = "assessment")

Data_Established_ToolLocationTime <- separate (Data_Established, col = "names", into = c("tooltype", "location", "year"), sep = " ")

Data_Established_ToolLocationTime|> group_by(tooltype,location,year)|>ggplot(aes(x = assessment, fill = year)) + geom_histogram() + facet_wrap(location ~ tooltype)

Data_Established_ToolLocationTime$assessment <- factor(Data_Established_ToolLocationTime$assessment, levels = c(0:3), labels = c("0","1","2","3"), ordered = TRUE)

Data_Established_23iNat <- Data_Established_ToolLocationTime[Data_Established_ToolLocationTime$year == "23" & Data_Established_ToolLocationTime$tooltype == "iNat",]

Data_Established_23iNat <- subset(Data_Established_23iNat, select = -Status)

Data_Established_23iNat$LabID <- as.factor (Data_Established_23iNat$LabID)

Data_Established_23 <- Data_Established_ToolLocationTime[Data_Established_ToolLocationTime$year == "23",]

Data_Established_23$location <- as.factor(Data_Established_23$location)

Data_Established_23$LabID <- as.factor(Data_Established_23$LabID)

summary(Data_Established$assessment)

**# Fit, predictions and plotting with clmm2**

Model_Hypothesis_Location_Established <- clmm2 (assessment ~ location, random = LabID, data=Data_Established_23iNat, Hess = TRUE, nAGQ =7)

summary(Model_Hypothesis_Location_Established)

pnorm(coef(summary(Model_Hypothesis_Location_Established))[,"z value"], lower.tail = FALSE)[-1:-3]

coef(summary(Model_Hypothesis_Location_Established))[, c(1,3)]

statsTable_Hypothesis_Location_Established <- cbind(coef(summary(Model_Hypothesis_Location_Established))[, c(1,3)],pnorm(coef (summary(Model_Hypothesis_Location_Established))[,"z value"], lower.tail = FALSE))

colnames(statsTable_Hypothesis_Location_Established)[3] <- "PR > Z || PR < Z"

p_Location_Established <- cbind(Data_Established_23iNat, pred = predict (Model_Hypothesis_Location_Established, newdata = Data_Established_23iNat))

p_Location_Established$assessment <- as.factor(p_Location_Established$assessment)

plot1 <- ggplot(p_Location_Established, aes(x = location, y = pred, fill = assessment)) + geom_point(shape = 21, size =3, color = "black") + scale_fill_manual(name = "Assessment", values = c("0"= "#000000", "1" = "#808080", "2" = "#D3D3D3", "3" = "#FFFFFF"), labels = c("0"= "Accurate", "1"= "Species-level error", "2"= "Genus-level error", "3"= "Family-level error")) + labs(title = "Established", x = "Location", y = "Predicted probabilities (%)") + theme(axis.text.x = element_text(size = 11, color = "black"), axis.text.y = element_text(size = 11, color = "black"), axis.title.x = element_text(size = 12, color = "black"), axis.title.y = element_text(size = 12, color = "black", margin = margin(r = 10)), panel.background = element_rect(fill = "white"), panel.border = element_rect(color = "black", fill = NA), panel.grid.major = element_blank(), panel.grid.minor = element_blank()) + scale_y_continuous(limits=c(0.00, 1.00), breaks = seq (0.00, 1.00, by = 0.20))

**### Predictor 1b - Outsider Plants**

**# Data Organization**

Data_Outsider_long_format <- read_excel("GB AI Project - Data long format.xlsx", sheet = "Outsider")

colnames (Data_Outsider_long_format)

Data_Outsider <- pivot_longer(Data_Outsider_long_format, cols = c(`iNat OTT 23`,`iNat ON 23`,`iNat NL 23`), names_to = "names", values_to = "assessment")

Data_Outsider_Location <- separate (Data_Outsider, col = "names", into = c("tooltype", "location", "year"), sep = " ")

Data_Outsider_Location|> group_by(location)|>ggplot(aes(x = assessment)) + geom_histogram() + facet_wrap(location ~ .)

Data_Outsider_Location <- drop_na (Data_Outsider_Location)

Data_Outsider_Location$assessment <- factor(Data_Outsider_Location$assessment, levels = c(0:3), labels = c("0","1","2","3"), ordered = TRUE)

Data_Outsider_Location$CatalogID <- as.factor(Data_Outsider_Location$CatalogID)

**# Fit, predictions and plotting with clmm2**

Model_Hypothesis_Location_Outsider <- clmm2 (assessment ~ location, random = CatalogID, data = Data_Outsider_Location, Hess = TRUE, nAGQ=7)

summary(Model_Hypothesis_Location_Outsider)

pnorm(coef(summary(Model_Hypothesis_Location_Outsider))[,"z value"],lower.tail = FALSE)[-1:-3]

coef(summary(Model_Hypothesis_Location_Outsider))[, c(1,3)]

statsTable_Hypothesis_Location_Outsider <- cbind(coef(summary(Model_Hypothesis_Location_Outsider))[, c(1,3)],pnorm(coef(summary(Model_Hypothesis_Location_Outsider))[,"z value"], lower.tail = FALSE))

colnames(statsTable_Hypothesis_Location_Outsider)[3] <- "PR > Z || PR < Z"

p_Location_Outsider <- cbind(Data_Outsider_Location, pred = predict (Model_Hypothesis_Location_Outsider, newdata = Data_Outsider_Location))

**# Fit, predictions and plotting with polr**

Model_Hypothesis_Location_Outsider_polr <- polr(assessment ~ location, data = Data_Outsider_Location, Hess = TRUE)

summary(Model_Hypothesis_Location_Outsider_polr)

pnorm(coef(summary(Model_Hypothesis_Location_Outsider_polr))[,"t value"], lower.tail = FALSE)[-1:-3]

coef(summary(Model_Hypothesis_Location_Outsider_polr))[, c(1,3)]

statsTable_Hypothesis_Location_Outsider_polr <- cbind(coef(summary(Model_Hypothesis_Location_Outsider_polr))[, c(1,3)],pnorm(coef (summary(Model_Hypothesis_Location_Outsider_polr))[,"t value"], lower.tail = FALSE))

colnames(statsTable_Hypothesis_Location_Outsider_polr)[3] <- "PR > Z || PR < Z"

p_Location_Outsider_polr <- cbind(Data_Outsider_Location, predict(Model_Hypothesis_Location_Outsider_polr, type = "probs"))

p_Location_Outsider_polr <- melt(p_Location_Outsider_polr, id.vars = c("CatalogID", "Species", "tooltype", "location", "year", "assessment"), value.name = "Probability")

plot2 <- ggplot(p_Location_Outsider_polr, aes(x = location, y = Probability , fill = variable)) + geom_point(shape = 21, size =3, color = "black") + scale_fill_manual(name = "Assessment", values = c("0"= "#000000", "1" = "#808080", "2" = "#D3D3D3", "3" = "#FFFFFF"), labels = c("0"= "Accurate", "1"= "Species-level error", "2"= "Genus-level error", "3"= "Family-level error")) + labs(title = "Outsider", x = "Location", y = "Predicted probabilities (%)") + scale_y_continuous(limits=c(0.00, 1.00), breaks = seq (0.00, 1.00, by = 0.20)) + theme(axis.text.x = element_text(size = 11, color = "black"), axis.text.y = element_text(size = 11, color = "black"), axis.title.x = element_text(size = 12, color = "black"), axis.title.y = element_text(size = 12, color = "black", margin = margin(r =10)), panel.background = element_rect(fill = "white"), panel.border = element_rect(color = "black", fill = NA), panel.grid.major = element_blank(), panel.grid.minor = element_blank())

**### Predictor 2 - Family**

**# Fit, predictions and plotting with clmm2**

Model_Hypothesis_Family_Established <- clmm2 (assessment ~ Family * tooltype, random = LabID, data=Data_Established_23, Hess = TRUE, nAGQ =7)

summary(Model_Hypothesis_Family_Established)

pnorm(coef(summary(Model_Hypothesis_Family_Established))[,"z value"],lower.tail = FALSE)[-1:-3]

coef(summary(Model_Hypothesis_Family_Established))[, c(1,3)]

statsTable_Hypothesis_Family_Established <- cbind(coef(summary(Model_Hypothesis_Family_Established))[, c(1,3)],pnorm(coef(summary(Model_Hypothesis_Family_Established))[,"z value"], lower.tail = FALSE))

colnames(statsTable_Hypothesis_Family_Established)[3] <- "PR > Z || PR < Z"

p_Plant_Family_Established <- cbind(Data_Established_23, pred = predict (Model_Hypothesis_Family_Established, newdata = Data_Established_23))

plot3 <- ggplot(p_Plant_Family_Established, aes(x = tooltype, y = pred, fill = assessment)) + geom_point(shape = 21, size =3, color = "black") + scale_fill_manual(name = "Assessment", values = c("0"= "#000000", "1" = "#808080", "2" = "#D3D3D3", "3" = "#FFFFFF"), labels = c("0"= "Accurate", "1"= "Species-level error", "2"= "Genus-level error", "3"= "Family-level error")) + facet_wrap(~Family) + labs(title = "Family", x = "Tool type", y = "Predicted probabilities (%)") + scale_y_continuous(limits=c(0.00, 1.00), breaks = seq (0.00, 1.00, by = 0.20)) + theme(strip.background = element_rect(fill = "#ffffff", color = "#000000", size=1), strip.text = element_text(size = 12, margin = margin(t=10, b=10)), axis.text.x = element_text(size = 11, color = "black"), axis.text.y = element_text(size = 11, color = "black"), axis.title.x = element_text(size = 12, color = "black"), axis.title.y = element_text(size = 12, color = "black", margin = margin (r=10)), panel.background = element_rect(fill = "white"), panel.border = element_rect(color = "black", fill = NA), panel.grid.major = element_blank(), panel.grid.minor = element_blank())

**### Predictor 3 - PlantParts**

**# Data organization**

Data_Plant_Parts_Established <- read_excel("GB AI Project - Data long format.xlsx", sheet = "PlantParts ")

colnames(Data_Plant_Parts_Established)

Data_Plant_Parts_Established <- pivot_longer(Data_Plant_Parts_Established, cols = c(`Leaves`, `Inflorescence`, `Whole Plant`), names_to = "plantparts", values_to = "assessment")

Data_Plant_Parts_Established|> group_by(plantparts)|>ggplot(aes(x = assessment)) + geom_histogram() + facet_wrap(plantparts ~ .)

Data_Plant_Parts_Established$assessment <- factor(Data_Plant_Parts_Established$assessment, levels = c(0:3), labels = c("0","1","2","3"), ordered = TRUE)

Data_Plant_Parts_Established$LabID <- as.factor(Data_Plant_Parts_Established$LabID)

**# Fit, predictions and plotting with clmm2**

Model_Hypothesis_Plant_Parts_Established <- clmm2 (assessment ~ plantparts, random = LabID, data=Data_Plant_Parts_Established, Hess = TRUE, nAGQ=7)

summary(Model_Hypothesis_Plant_Parts_Established)

pnorm(coef(summary(Model_Hypothesis_Plant_Parts_Established))[,"z value"],lower.tail = FALSE)[-1:-3]

coef(summary(Model_Hypothesis_Plant_Parts_Established))[, c(1,3)]

statsTable_Hypothesis_Plant_Parts_Established <- cbind(coef(summary(Model_Hypothesis_Plant_Parts_Established))[, c(1,3)],pnorm(coef(summary(Model_Hypothesis_Plant_Parts_Established))[,"z value"], lower.tail = FALSE))

colnames(statsTable_Hypothesis_Plant_Parts_Established)[3] <- "PR > Z || PR < Z"

p_Plant_Parts_Established <- cbind(Data_Plant_Parts_Established, pred = predict (Model_Hypothesis_Plant_Parts_Established, newdata = Data_Plant_Parts_Established))

plot4 <- ggplot(p_Plant_Parts_Established, aes(x = plantparts, y = pred, fill = assessment)) + geom_point(shape = 21, size =3, color = "black") + scale_fill_manual(name = "Assessment", values = c("0"= "#000000", "1" = "#808080", "2" = "#D3D3D3", "3" = "#FFFFFF"), labels = c("0"= "Accurate", "1"= "Species-level error", "2"= "Genus-level error", "3"= "Family-level error")) + labs(title = "Plant parts", x = "Plant Parts", y = "Predicted probabilities (%)") + scale_y_continuous(limits=c(0.00, 1.00), breaks = seq (0.00, 1.00, by = 0.20)) + theme(axis.text.x = element_text(size = 11, color = "black"), axis.text.y = element_text(size = 11, color = "black"), axis.title.x = element_text(size = 12, color = "black"), axis.title.y = element_text(size = 12, color = "black", margin = margin (r = 10)), panel.background = element_rect(fill = "white"), panel.border = element_rect(color = "black", fill = NA), panel.grid.major = element_blank(), panel.grid.minor = element_blank())

**### Predictor 4 – Status**

**# Fit, predictions and plotting with clmm2**

Model_Hypothesis_Status_Established <- clmm2 (assessment ~ Status * tooltype, random = LabID, data=Data_Established_23, Hess = TRUE, nAGQ =7)

summary(Model_Hypothesis_Status_Established)

pnorm(coef(summary(Model_Hypothesis_Status_Established))[,"z value"], lower.tail = FALSE)[-1:-3]

coef(summary(Model_Hypothesis_Status_Established))[, c(1,3)]

statsTable_Hypothesis_Status_Established <- cbind(coef(summary(Model_Hypothesis_Status_Established))[, c(1,3)],pnorm(coef (summary(Model_Hypothesis_Status_Established))[,"z value"], lower.tail = FALSE))

colnames(statsTable_Hypothesis_Status_Established)[3] <- "PR > Z || PR < Z"

p_Status_Established <- cbind(Data_Established_23, pred = predict (Model_Hypothesis_Status_Established, newdata = Data_Established_23))

plot5 <- ggplot(p_Status_Established, aes(x = tooltype, y = pred, fill = assessment)) + geom_point(shape = 21, size =3, color = "black") + scale_fill_manual(name = "Assessment", values = c("0"= "#000000", "1" = "#808080", "2" = "#D3D3D3", "3" = "#FFFFFF"), labels = c("0"= "Accurate", "1"= "Species-level error", "2"= "Genus-level error", "3"= "Family-level error")) + facet_wrap(~Status) + labs(title = "Distribution Status", x = "Tool type", y = "Predicted probabilities (%)") + scale_y_continuous(limits=c(0.00, 1.00), breaks = seq (0.00, 1.00, by = 0.20)) + theme(strip.background = element_rect(fill = "#ffffff", color = "#000000", size=1), strip.text = element_text(size = 12, margin = margin(t=10, b=10)), axis.text.x = element_text(size = 11, color = "black"), axis.text.y = element_text(size = 11, color = "black"), axis.title.x = element_text(size = 12, color = "black"), axis.title.y = element_text(size = 12, color = "black", margin = margin (r = 10)), panel.background = element_rect(fill = "white"), panel.border = element_rect(color = "black", fill = NA), panel.grid.major = element_blank(), panel.grid.minor = element_blank())

**### Combining plots**

plot_Location_Combined <- plot1 + plot2 + plot_layout(ncol = 2, guides = "collect")

print(plot_Location_Combined)

plot_Family_Status_Combined <- plot3 + plot5 + plot_layout(ncol = 2, guides = "collect")

print(plot_Family_Status_Combined)

**### Proportional Odd Assumption Testing**

**# Predictor 1a - Location Established Plants**

Clmm_Hypothesis_Location_Established <- clmm(assessment ~ location + (1|LabID), data= Data_Established_23iNat, Hess = TRUE, nAGQ =7)

summary(Clmm_Hypothesis_Location_Established)

emmeans_Location_Established <- emmeans (Clmm_Hypothesis_Location_Established, list(pairwise~location))

summary(emmeans_Location_Established)

**# Predictor 1b - Location Outsider Plants**

Clmm_Hypothesis_Location_Outsider <- clmm(assessment ~ location + (1|CatalogID), data= Data_Outsider_Location, Hess = TRUE, nAGQ =7)

summary(Clmm_Hypothesis_Location_Outsider)

emmeans_Location_Outsider <- emmeans (Clmm_Hypothesis_Location_Outsider, list(pairwise~location))

summary(emmeans_Location_Outsider)

brant(Model_Hypothesis_Location_Outsider_polr)

**# Predictor 2 – Family * Tool type**

Clmm_Hypothesis_Family_Established <- clmm(assessment ~ Family * tooltype + (1|LabID), data= Data_Established_23, Hess = TRUE, nAGQ =7)

summary(Clmm_Hypothesis_Family_Established)

emmeans_Family <- emmeans (Clmm_Hypothesis_Family_Established, list(pairwise~Family, pairwise~tooltype))

summary(emmeans_Family)

**# Predictor 3 – Plant Parts**

Clmm_Hypothesis_Plant_Parts_Established <- clmm(assessment ~ plantparts + (1|LabID), data= Data_Plant_Parts_Established, Hess = TRUE, nAGQ =7)

summary(Clmm_Hypothesis_Plant_Parts_Established)

emmeans_PlantParts <- emmeans (Clmm_Hypothesis_Plant_Parts_Established, list(pairwise~plantparts))

summary(emmeans_PlantParts)

**# Predictor 4 – Status * Tool type**

Clmm_Hypothesis_Status_Established <- clmm(assessment ~ Status * tooltype + (1|LabID), data= Data_Established_23, Hess = TRUE, nAGQ =7)

summary(Clmm_Hypothesis_Status_Established)

emmeans_Status <- emmeans (Clmm_Hypothesis_Status_Established, list(pairwise~Status, pairwise~tooltype))

summary(emmeans_Status)
